# The Significance of Metastasectomy in Patients with Metastatic Renal Cell Carcinoma in the Era of Targeted Therapy

**DOI:** 10.1155/2015/176373

**Published:** 2015-10-11

**Authors:** Xiaoteng Yu, Bing Wang, Xuesong Li, Gang Lin, Cuijian Zhang, Yang Yang, Dong Fang, Yi Song, Zhisong He, Liqun Zhou

**Affiliations:** ^1^Department of Urology, Peking University First Hospital, Institute of Urology, National Urological Cancer Center, Peking University, Beijing 100034, China; ^2^Department of Orthopedics, Peking University First Hospital, Beijing 100034, China; ^3^Department of Thoracic Surgery, Peking University First Hospital, Beijing 100034, China

## Abstract

*Objective.* To investigate the efficacy of surgery in the treatment of metastatic renal cell carcinoma (mRCC) and to identify prognostic factors.* Methods.* A single center retrospective study of 96 patients with mRCC from December 2004 to August 2013.* Results.* The median follow-up time was 45 months. Thirty-one (32.3%) of the patients received complete resection of metastatic sites, 11 (11.5%) of the patients underwent incomplete resection of metastatic sites, and 54 (56.3%) of the patients received no surgery. In the univariate Kaplan-Meier analysis, the median overall survival times of the three groups were 52 months, 16 months, and 22 months, respectively (*p* < 0.001). The difference in the overall survival time was statistically significant between complete resection and no surgery groups (HR = 0.43, *p* = 0.009), while there was no significant difference between the incomplete metastasectomy and no surgery groups (HR = 1.80, *p* = 0.102). According to the multivariate Cox regression analysis, complete metastasectomy (HR = 0.49, *p* = 0.033), T stage > 3 (HR = 1.88, *p* = 0.015), disease free interval <12 months (HR = 2.34, *p* = 0.003), and multiorgan involvement (HR = 2.00, *p* = 0.011) were significant prognostic factors.* Conclusion.* In the era of targeted therapy, complete metastasectomy can improve overall survival. Complete metastasectomy, T stage > 3, disease free interval <12 months, and multiorgan involvement are independent prognostic factors.

## 1. Introduction

Kidney cancer accounts for approximately 2-3% of all the adult malignancies, and the incidence is increasing [1]. Despite the advancements in early diagnosis, 20–30% of patients present with synchronous metastatic renal cell carcinoma (mRCC) [2]. Approximately one-third of patients experience disease relapse as either a local recurrence or distant metastasis, after the primary surgery for the renal tumors [3, 4]. Metastatic renal cell carcinoma is associated with a poor prognosis and a 5-year survival rate not more than 10% [5]. Before the emergence of targeted therapy, immunotherapy was the main therapeutic option. However, a low response rate and high incidence of adverse events make this option only suitable for a specific subset of patients [6, 7].

Targeted drugs, such as sunitinib, sorafenib, everolimus, and bevacizumab, have improved the tumor response rate and changed the treatment algorithms of mRCC in recent years [8–10]. However, the complete response rate was rather low, and none of the drugs were curative [11, 12]. We cannot ignore the significance of metastasectomy in regard to curative intent. Although previous reports are promising, the role of metastasectomy in an era of targeted therapy is an actively researched field. Thus, we performed a retrospective analysis of 107 patients in our center to elucidate the significance of metastasectomy in the treatment of mRCC. Our main objective was to investigate the impact of metastasectomy on survival time and to identify prognostic factors related to survival.

## 2. Materials and Methods

After ethical committee approval, a total of 130 patients with metastatic renal cell carcinoma treated in Peking University First Hospital between December 2004 and August 2013 were retrospectively included in our study. The diagnoses of renal cell carcinoma were made based on histopathological evaluations of the specimens acquired by previous nephrectomy or renal biopsy. All the 130 patients were with oligometastasis and a Karnofsky Performance Scale (KPS) not less than 80. The metastatic sites were defined by computed tomography (CT), magnetic resonance imaging (MRI), and positron emission tomography (PET) or confirmed by pathological outcomes of the metastasectomy. 11 of the 130 patients did not have previous nephrectomy and were excluded from the study. Another 23 patients were excluded due to incomplete data concerning survival time, pathology, metastatic sites, and detailed record of surgery. Among the 23 patients, 16 patients did not receive metastasectomy, 1 patient received incomplete metastasectomy, 5 patients underwent complete metastasectomy, and 1 patient lacked the detailed record of surgery. And finally we identified 96 patients for the definitive analysis.

We retrospectively collected the clinical and pathologic characteristics of the patients, including gender, age at first metastasis development, targeted therapy, pathology, disease-free interval (DFI), sites of metastases, number of organs with metastases, and the surgical margins of the metastasectomy. The primary nephrectomy was either a partial nephrectomy or a radical nephrectomy. The different nephrectomies were decided on a case-by-case basis. The 2004 WHO classification of renal tumors was used when evaluating the pathology [13]. Complete metastasectomy was defined as resection of all the metastases, while incomplete metastasectomy was defined as resection of some but not all the metastases. The DFI was defined as the period between the primary diagnosis of the renal tumor and the first occurrence of the metastases.

After the initial treatment, there was a follow-up appointment every 6 months, during which an abdominal ultrasound, chest X-ray, or CT and a routine blood test were performed and evaluated. Also, medical histories were collected and necessary physical examinations were taken. A bone scan and MRI of the brain were used in cases of overt symptoms. 51 patients had overt symptoms indicating metastasis which were further confirmed by imaging studies. Repeat metastasectomies were indicated in 5 patients with relapse of the disease who were in otherwise good condition.

We compared clinical and pathological characteristics among complete metastasectomy, incomplete metastasectomy, and no metastasectomy groups, using Pearson's chi-square for categorical variables. The primary endpoint was overall survival (OS), which was calculated from the first metastasis development to death or the time of last follow-up. We used a Kaplan-Meier method to estimate the OS. Univariable Kaplan-Meier methods with log rank tests and univariable Cox regression methods were performed to compare the survival difference between the groups. A multivariable Cox regression model was used to identify the prognostic factors of OS. All of the statistical analyses were made using SPSS 20.0 (IBM Corporation, New York, USA), and *p* value < 0.05 was considered to be statistically significant.

## 3. Results

### 3.1. Patients Characteristics

Our study comprised 96 mRCC patients, including 78 males (81.3%) and 18 females (18.7%). The median age was 57 years (range: 17–79 years). All of the patients had undergone a prior nephrectomy for a primary tumor, and the majority of the patients (75.0%) received targeted therapy. 16 patients underwent interferon as adjuvant therapy after primary nephrectomy, and 10 patients received interleukin-2, while 2 patients received chemotherapy. Clear cell carcinoma was the dominant pathology. A small percentage of the patients exhibited clear cell carcinoma with sarcomatoid dedifferentiation. Lung and bone were the most common metastatic sites. 63.5% of the patients had only one organ involvement. More than half of the patients had a relatively short DFI. For the treatment of the metastatic lesions, 31 patients received a complete resection and 11 patients underwent an incomplete resection, while 54 patients received no surgery and opted for targeted therapy. 25 patients in the complete resection group had solitary lesion. No neoadjuvant or immediate adjuvant targeted therapy was used in the metastasectomy group. Targeted therapy was only administered in case of inoperable progressive disease in this group. Detailed clinical and pathological characteristics of the patients are shown in Table 1.

The clinical and pathological differences between these groups were not significant concerning gender (*p* = 0.262), age (*p* = 0.953), clear cell carcinoma or nonclear cell carcinoma (*p* = 0.217), disease-free interval (*p* = 0.154), T stage (*p* = 0.979), grade (*p* = 0.155), and lymph node involvement (*p* = 0.111). But there were significant differences concerning multiorgan involvement or single organ involvement between these groups (*p* = 0.042) (Table 2).

All of the patients in the no resection group were given targeted drugs; the efficacy evaluation showed that 1 (1.8%) patient achieved complete responses (CR), 13 (24.1%) patients reached partial responses (PR), 38 (70.4) patients experienced stable disease (SD), and 2 (3.7%) patients developed progressive disease. Efficacy was defined according to the Response Evaluation Criteria in Solid Tumors (RECIST) criteria [14].

### 3.2. Overall Survival by Kaplan-Meier Analysis

**Figure 1:**
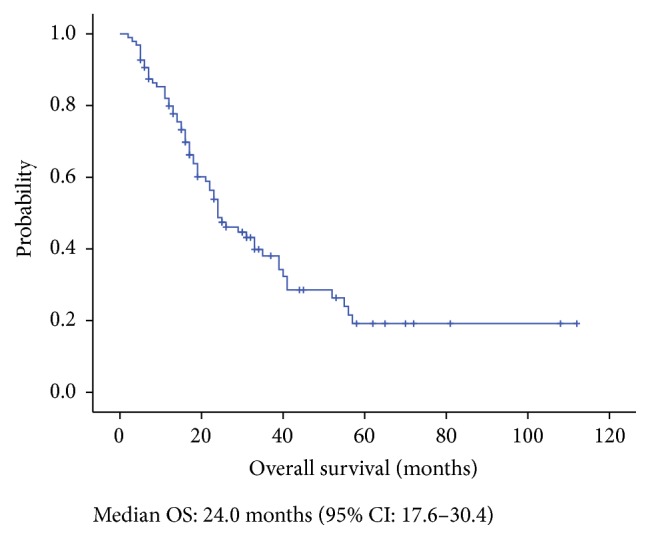
Overall survival (median OS: 24.0 months).

The median follow-up time of the 96 patients was 45 months (range: 2–112 months). Seventy patients (63.5%) died prior to the last follow-up. Considering that only 2 patients died of non-cancer-related causes (atrial fibrillation), we decided to use overall survival as the primary endpoint. According to the Kaplan-Meier analysis, the median OS of the 96 patients was 24.0 months (95% CI: 17.6–30.4 months) (Figure 1).

### 3.3. Univariable Analysis by Kaplan-Meier

**Figure 2:**
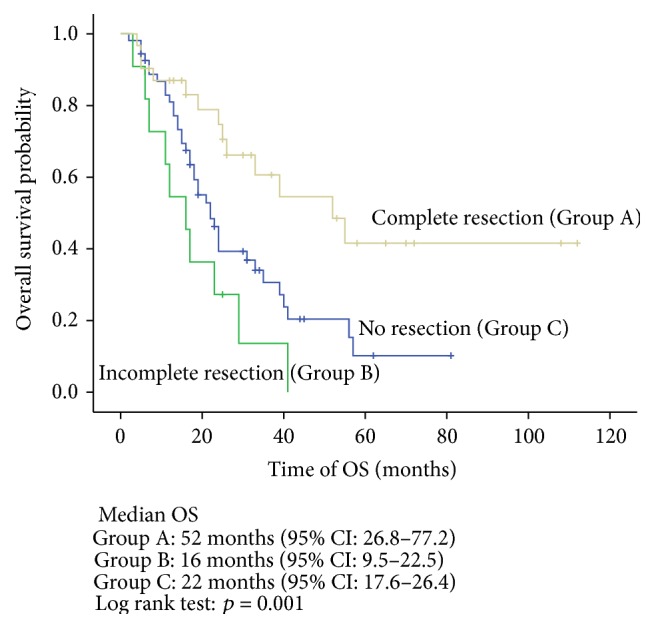
Overall survival stratified by completeness of resection (Group A: complete resection; Group B: incomplete resection; Group C: no resection).

**Figure 3:**
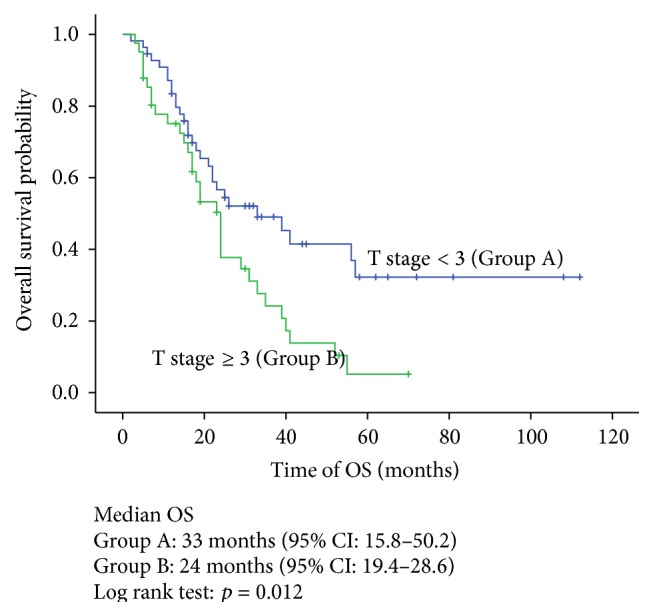
Overall survival stratified by T stage (Group A: T stage < 3; Group B: T stage ≥ 3).

**Figure 4 fig4:**
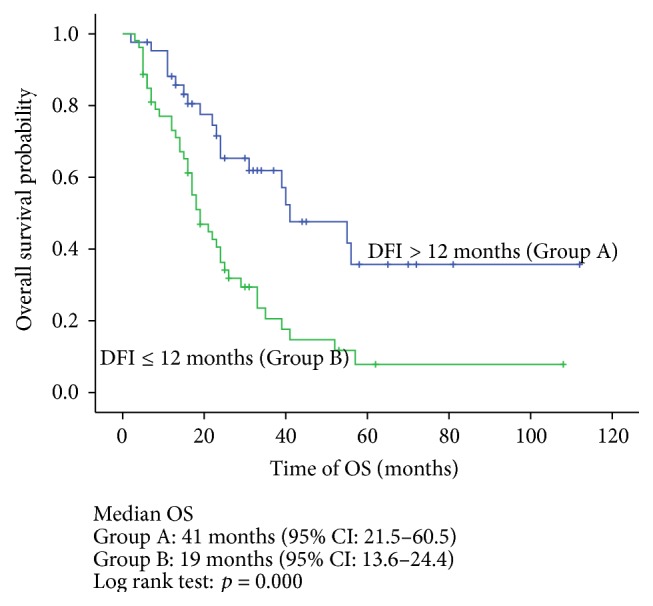
Overall survival stratified by DFI (Group A: DFI > 12 months; Group B: DFI ≤ 12 months.).

The Kaplan-Meier univariate analysis indicated that the median OS in the complete resection, incomplete resection, and no resection groups was 52 months, 16 months, and 22 months, respectively. The difference between the groups was statistically significant (*p* = 0.001) (Figure 2). Meanwhile, T stage ≥ 3 (*p* = 0.012), the ≤12-month DFI (*p* = 0.000), and multiorgan involvement (*p* = 0.001) had adverse effects on OS (Figures 3–5). Other potential prognostic factors used in our analysis included gender, year, targeted therapy, pathological characteristics, lymph node involvement, and metastatic sites. However, none of these factors were statistically significant (data not shown).

Also, we performed a subgroup analysis of all the patients who underwent metastasectomy but did not show a significant difference between the targeted therapy group and nontargeted therapy group (median OS: 26 months versus 33 months, *p* = 0.706).

### 3.4. Multivariable Analysis by Cox Regression

When using Cox regression univariate analysis, there was a significant difference between the complete metastasectomy and no resection groups (HR = 0.43, *p* = 0.009). With this analysis, there was no significant difference between the incomplete metastasectomy and no resection groups (HR = 1.80, *p* = 0.102). Also, T stage ≥ 3 (HR = 1.88, *p* = 0.015), DFI ≤ 12 months (HR = 2.59, *p* = 0.001), and multiorgan involvement (HR = 2.25, *p* = 0.002) were associated with poor OS (Table 3).

Based on the multivariate Cox regression analysis, a complete metastasectomy was still a favorable predictor of OS (HR = 0.49, *p* = 0.033), while an incomplete metastasectomy seemed to decrease the overall survival compared with no resection for metastatic sites, though the influence was not statistically significant (HR = 1.35, *p* = 0.418). The influence of T stage ≥ 3 (HR = 1.89, *p* = 0.018), the DFI ≤ 12 months (HR = 2.34, *p* = 0.003), and multiorgan involvement (HR = 2.00, *p* = 0.011) on OS was still present (Table 4).

## 4. Discussion

The robust clinical responses of targeted therapy have revitalized the treatment of metastatic renal cell carcinoma. However, the long-term survival for patients with renal cell carcinoma remains low. Surgery as part of a multimodal treatment of mRCC is a potential way to improve the long-term survival. Prospective randomized trials have proven the importance of cytoreductive nephrectomy in the cytokine era [15]. The literature in recent years also favors cytoreductive nephrectomy to increase the overall survival in this era of targeted therapy [16, 17].

In 1939, Barney and Churchill reported the first metastasectomy performed on a 55-year-old woman with a solitary pulmonary metastasis. After the resection, the prognosis was quite promising. Since then, increasing evidence has shown that resection of metastatic sites benefits the OS or the 5-year survival rate in appropriately selected patients. Alt et al. investigated 887 mRCC patients and found better CSS in patients with a complete metastasectomy (4.8 years versus 1.3 years, *p* < 0.001). Furthermore, a subgroup analysis of the lung-only metastases or non-lung-only metastases groups and synchronous or asynchronous multiple metastases groups also showed an improvement in the CSS and the 5-year survival rate in favor of a complete metastasectomy [18]. Naito et al. reviewed 566 mRCC patients in a multicenter retrospective study and reported a median OS time of 109.8 months and 31.9 months in the complete metastasectomy and incomplete metastasectomy groups, respectively, which was statistically significant. The authors also demonstrated that incomplete resection of metastases adversely affected the OS using multivariate analysis [19].

Our results concerning the impact of metastasectomy on OS were consistent with previous studies. The data in the present study showed that the OS in the complete metastasectomy, incomplete metastasectomy, and no resection groups was 52, 16, and 22 months, respectively. The results showed better OS in favor of a complete resection of metastases compared with no resection. Meanwhile, incomplete metastasectomy was associated with worse OS. Although the difference was not statistically significant, the OS in the incomplete resection group was shorter than in the no resection group.

Several factors may have led to the decreased OS in the incomplete resection and no resection groups. First, the tumors in the incomplete resection group have more metastatic sites than the other two groups, making a complete resection of all the metastatic sites even impossible. Second, the tumors in the incomplete resection group may have been more invasive, which made a complete resection rather difficult during the surgery. Third, some of the patients who underwent a metastasectomy were distressed by pain or loss of function; therefore, surgery in these situations was not performed with a curative intent but with palliative intent and resulted in an incomplete resection.

Despite the lack of clear improvement in OS and possible postoperative complications, metastasectomy may help to ameliorate pain and improve functional recovery. Thus, an incomplete resection has a role in improving quality of life if the metastatic sites are in weight-bearing bones, causes severe pain, or compromises neurological function [20]. We recommend performing a metastasectomy only after a full evaluation, including determining the resectability of the tumor and the patients' personal condition.

In addition to resectability, several potential prognostic factors should be evaluated. There is no consensus regarding the standardized tools that help assure the best candidates for a metastasectomy, and most of the evidence is based on retrospective studies. In a 64-patient study, Hofmann et al. reported that in patients receiving a pulmonary metastasectomy the synchronous metastases had a worse 5-year survival rate compared with metachronous metastases (0% versus 43.7%), respectively, suggesting that the DFI may have an impact on the survival of patients who underwent metastasectomy. However, the sample size was relatively small, and the metastases were exclusively in the lung [21]. Tosco et al. performed a multicenter retrospective analysis of 109 patients and identified four independent adverse factors on prognosis: T stage of the primary tumor ≥3, Fuhrman grade ≥3, disease-free interval ≤12 months, and multiorgan metastases [22]. The data in our study were in line with previous reports and also indicated that T stage ≥ 3 (HR = 1.88, *p* = 0.015), disease-free interval ≤12 months (HR = 2.34, *p* = 0.003), and multiorgan involvement (HR = 2.00, *p* = 0.011) were associated with poor OS.

The primary limitation of our study is that it is a retrospective study. There were biases we were unable to avoid. Patients selected for complete metastasectomy may have had better ECOG PS, lower risk MSKCC [23], or Heng group [24] and therefore had better survival. Some of the patients who underwent an incomplete resection were only attempting to improve their quality of life by alleviating their pain or recovering function. In these cases, the procedure was not intended to be curative. Also, the number of patients who underwent incomplete metastasectomy was small, and the results merit further investigation. Unfortunately, the lack of data regarding these aspects made this analysis difficult to carry out. Due to incomplete records on the specific targeted therapy usage, such as duration of medication or drug dosage, we were unable to incorporate detailed information in our analysis despite their potential effects on survival.

In conclusion, our study shows that complete metastasectomy can improve OS in the era of targeted therapy, whereas incomplete resection did not. A T stage ≥ 3, DFI ≤ 12 months, and multiorgan involvement are independent adverse prognostic factors for OS.

## Figures and Tables

**Figure 5:**
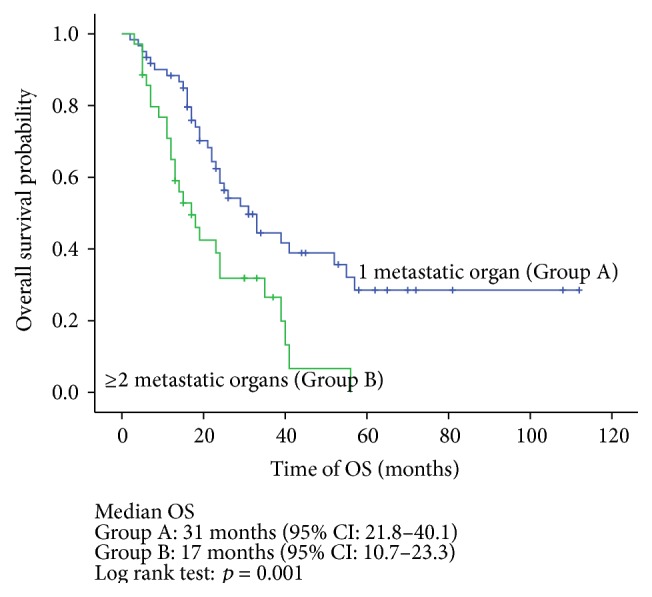
Overall survival stratified by number of metastatic sites (Group A: 1 metastatic organ; Group B: ≥2 metastatic organs).

**Table 1:** Clinical and pathological characteristics.

Characteristics	Number	Percent (%)
Total patients	96	100.0
Gender		
Male	78	81.3
Female	18	18.7
Years ≥ 65		
Yes	26	27.1
No	70	72.9
Targeted therapy		
Sunitinib	35	36.5
Sorafenib	37	38.5
No	24	25.0
Pathology		
Clear cell	90	93.8
Papillary	5	5.2
Undifferentiated	1	1.0
Sarcomatoid dedifferentiation		
Yes	11	11.5
No	85	88.5
T		
T1-2	55	57.3
T3-4	41	42.7
N		
N0	87	90.6
N1-2	9	9.4
G		
G1-2	68	70.8
G3-4	28	29.2

Metastatic sites		
Lung	55	57.3
Bone	39	40.6
Liver	8	8.3
Brain	4	4.2
Adrenal gland	14	14.6
Retroperitoneal lymph node	13	13.5
Supradiaphragmatic lymph node	4	4.2
Others	7	7.3
Metastatic organ		
1	61	63.5
≥2	35	36.5
Disease-free interval		
≤12 months	53	55.2
>12 months	43	44.8
Metastasectomy		
Complete resection	31	32.3
Incomplete resection	11	11.5
No resection	54	56.3

**Table 2:** Difference of critical clinical and pathological characteristics between groups.

Characteristics	Number Complete metastasectomy	Number Incomplete metastasectomy	Number No metastasectomy	*p* value
Gender				
Male	28	9	41	0.262
Female	3	2	13	
Years				
65 or more	9	3	14	0.953
Less than 65	22	8	40	
Pathology				
Clear cell carcinoma	31	10	49	0.217
Others	0	1	5	
T				
T1-2	18	6	31	0.979
T3-4	13	5	23	
N				
N0	30	11	46	0.111
N1-2	1	0	8	
G				
G1-2	18	8	42	0.155
G3-4	13	3	12	
DFI				
12 months or less	17	9	27	0.154
More than 12 months	14	2	27	
Metastatic organ				
1	25	5	31	0.042^*∗*^
≥2	6	6	23	

^*∗*^Statistically significant.

**Table 3:** Univariate Cox regression analysis of prognostic factors of overall survival.

Parameter	Patients		Overall survival		*p* value
	*n*	Percent %	Median, mo	HR, 95% CI	
Overall	96	100	24		
Gender					
Male	78	81.3	25	1.25 (0.66–2.37)	0.490
Female	18	18.7	23	Reference	
Years ≥ 65					
Yes	26	27.1	23	1.34 (0.78–2.32)	0.293
No	70	72.9	24	Reference	
Targeted therapy					
Yes	72	75.0	24	1.29 (0.71–2.35)	0.406
No	24	25.0	33	Reference	
Clear cell carcinoma					
Yes	90	93.8	25	0.56 (0.22–1.41)	0.218
No	6	6.2	17	Reference	
Sarcomatoid dedifferentiation					
Yes	11	11.5	13	1.74 (0.85–3.53)	0.129
No	85	88.5	25	Reference	
T stage					
T ≥ 3	41	42.7	24	1.88 (1.13–3.13)	0.015^*∗*^
T < 3	55	57.3	33	Reference	
G grade					
G ≥ 3	28	29.2	24	1.39 (0.81–2.37)	0.231
G < 3	68	70.8	26	Reference	

N					
N1-2	9	9.4	24	0.83 (0.36–1.93)	0.660
N0	87	90.6	24	Reference	
Lung metastasis					
Yes	55	57.3	24	1.60 (0.94–2.73)	0.086
No	41	42.7	33	Reference	
Bone metastasis					
Yes	39	40.6	29	0.95 (0.57–1.58)	0.846
No	57	59.4	24	Reference	
Number of metastatic organs					
1	61	63.5	31	Reference	
≥2	35	36.5	17	2.25 (1.34–3.78)	0.002^*∗*^
Disease-free interval					
≤12 months	53	55.2	19	2.59 (1.50–4.48)	0.001^*∗*^
>12 months	43	44.8	41	Reference	
Metastasectomy					
Complete resection	31	32.3	52	0.43 (0.23–0.81)	0.009^*∗*^
Incomplete resection	11	11.5	16	1.80 (0.89–3.62)	0.102
No resection	54	56.3	22	Reference	

^*∗*^Statistically significant.

**Table 4:** Multivariate Cox regression analysis of prognostic factors of overall survival.

Covariates	HR	*p* value	95% CI
No resection	Reference		
Incomplete resection	1.35	0.418	0.65–2.77
Complete resection	0.49	0.033	0.25–0.94
T stage ≥ 3 (Yes versus No)	1.89	0.018	1.11–3.20
Multiorgan metastasis (Yes versus No)	2.00	0.011	1.17–3.41
DFI ≤ 12 months (Yes versus No)	2.34	0.003	1.33–4.12

## References

[1] Siegel R., Naishadham D., Jemal A. (2013). Cancer statistics, 2013. *CA Cancer Journal for Clinicians*.

[2] Staehler M., Haseke N., Schoeppler G., Stadler T., Gratzke C., Stief C. G. (2007). Modern therapeutic approaches in metastatic renal cell carcinoma. *EAU-EBU Update Series*.

[3] Cohen H. T., McGovern F. J. (2005). Renal-cell carcinoma. *The New England Journal of Medicine*.

[4] Mickisch G. H. J., Garin A., van Poppel H., de Prijck L., Sylvester R. (2001). Radical nephrectomy plus interferon-alfa-based immunotherapy compared with interferon alfa alone in metastatic renal-cell carcinoma: a randomised trial. *The Lancet*.

[5] Rini B. I., Campbell S. C., Escudier B. (2009). Renal cell carcinoma. *The Lancet*.

[6] Coppin C., Porzsolt F., Awa A., Kumpf J., Coldman A., Wilt T. (2005). Immunotherapy for advanced renal cell cancer. *The Cochrane Database of Systematic Reviews*.

[7] Bukowshi R. M. (2001). Cytokine therapy for metastatic renal cell carcinoma. *Seminars in Urologic Oncology*.

[8] Motzer R. J., Rini B. I., Bukowski R. M. (2006). Sunitinib in patients with metastatic renal cell carcinoma. *The Journal of the American Medical Association*.

[9] Escudier B., Eisen T., Stadler W. M. (2007). Sorafenib in advanced clear-cell renal-cell carcinoma. *The New England Journal of Medicine*.

[10] Escudier B., Pluzanska A., Koralewski P. (2007). Bevacizumab plus interferon alfa-2a for treatment of metastatic renal cell carcinoma: a randomised, double-blind phase III trial. *The Lancet*.

[11] Rini B. I. (2007). Vascular endothelial growth factor-targeted therapy in renal cell carcinoma: current status and future directions. *Clinical Cancer Research*.

[12] Rini B. I. (2009). Vascular endothelial growth factor-targeted therapy in metastatic renal cell carcinoma. *Cancer*.

[13] Lopez-Beltran A., Scarpelli M., Montironi R., Kirkali Z. (2006). 2004 WHO classification of the renal tumors of the adults. *European Urology*.

[14] Duffaud F., Therasse P. (2000). New guidelines to evaluate the response to treatment in solid tumors. *Bulletin du Cancer*.

[15] Flanigan R. C., Mickisch G., Sylvester R., Tangen C., Van Poppel H., Crawford E. D. (2004). Cytoreductive nephrectomy in patients with metastatic renal cancer: a combined analysis. *The Journal of Urology*.

[16] Zini L., Capitanio U., Perrotte P. (2009). Population-based assessment of survival after cytoreductive nephrectomy versus no surgery in patients with metastatic renal cell carcinoma. *Urology*.

[17] Abern M. R., Scosyrev E., Tsivian M., Messing E. M., Polascik T. J., Dudek A. Z. (2014). Survival of patients undergoing cytoreductive surgery for metastatic renal cell carcinoma in the targeted-therapy era. *Anticancer Research*.

[18] Alt A. L., Boorjian S. A., Lohse C. M., Costello B. A., Leibovich B. C., Blute M. L. (2011). Survival after complete surgical resection of multiple metastases from renal cell carcinoma. *Cancer*.

[19] Naito S., Kinoshita H., Kondo T. (2013). Prognostic factors of patients with metastatic renal cell carcinoma with removed metastases: a multicenter study of 556 patients. *Urology*.

[20] Ruutu M., Bono P., Taari K. (2008). Resection of renal cell cancer metastases: where do we stand in 2008?. *European Urology Supplements*.

[21] Hofmann H.-S., Neef H., Krohe K., Andreev P., Silber R.-E. (2005). Prognostic factors and survival after pulmonary resection of metastatic renal cell carcinoma. *European Urology*.

[22] Tosco L., Van Poppel H., Frea B., Gregoraci G., Joniau S. (2013). Survival and impact of clinical prognostic factors in surgically treated metastatic renal cell carcinoma. *European Urology*.

[23] Motzer R. J., Bacik J., Schwartz L. H. (2004). Prognostic factors for survival in previously treated patients with metastatic renal cell carcinoma. *Journal of Clinical Oncology*.

[24] Ko J. J., Xie W., Kroeger N. (2015). The International Metastatic Renal Cell Carcinoma Database Consortium model as a prognostic tool in patients with metastatic renal cell carcinoma previously treated with first-line targeted therapy: a population-based study. *The Lancet Oncology*.

